# Biological Drivers of IMiD‐Induced Peripheral Neuropathy in Multiple Myeloma

**DOI:** 10.1002/jha2.70323

**Published:** 2026-06-02

**Authors:** Sina A. Beer, Richard Houlston, Martin F. Kaiser

**Affiliations:** ^1^ Division of Genetics and Epidemiology The Institute of Cancer Research London UK

**Keywords:** drug‐induced toxicity, genetic susceptibility, IMiD‐induced toxicity, multiple myeloma, peripheral neuropathy

## Abstract

The introduction of immunomodulatory drugs (IMiDs), such as thalidomide, lenalidomide and pomalidomide, have led to significant improvements in multiple myeloma (MM) treatment. Many patients now live 10 years or longer with their disease, making treatment‐related side effects increasingly important. Peripheral neuropathy (PN) is a common side effect of IMiD therapy, with a substantial impact on patients’ quality of life, and no preventive or targeted therapeutic strategies are currently available. This review explores pathomechanisms and genetic factors likely underlying IMiD‐induced PN, considering oxidative stress, microvascular changes, immune‐mediated injury and neuroinflammation, neurotrophin dysregulation, as well as heritable susceptibility. Particular focus is given to potential mechanisms explaining the distinct neurotoxicity profile of thalidomide compared to second‐generation IMiDs. In this context, the review highlights the need for standardized clinical trial assessments, larger genetic studies and robust preclinical models to elucidate causality and manage this complex toxicity effectively.

**Trial Registration**: The authors have confirmed clinical trial registration is not needed for this submission.

## 1 Background

Therapeutic advancements in multiple myeloma (MM) have significantly prolonged patient survival [1], with IMiDs, including thalidomide (Thal), lenalidomide (Len) and pomalidomide (Pom), serving as cornerstones across all patient groups [2, 3]. These glutarimide derivatives bind primarily to cereblon (CRBN), altering its substrate specificity and promoting proteasomal degradation of the transcription factors IKZF1 and IKZF3 in MM cells [4, 5, 6]. Beyond their direct tumoricidal actions, IMiDs are thought to exert immunomodulatory, anti‐proliferative and anti‐angiogenic effects by modulating NFκB signalling, cytokines (e.g., TNF‐α and IL‐6) and growth factors such as VEGF, while also enhancing T‐cell activity [7, 8, 9, 10]. Compared to Thal, second‐ and next‐generation IMiDs (so‐called CELMoDs) demonstrate higher potency and greater target specificity [11, 12, 13], likely contributing to their distinct side‐effect profiles. IMiDs are administered orally with high bioavailability (> 90%) and short half‐lives of 3–7 h [14]. Thal and Pom undergo partial hepatic metabolism [15, 16], while Len is primarily renally cleared [17]. Notably, IMiDs can cross the blood–nerve barrier [18]. The more lipophilic Thal achieves cerebrospinal fluid (CSF) concentrations up to 50% of its plasma levels, compared to around 10% for Len [19].

Despite their therapeutic efficacy, IMiD‐induced peripheral neuropathy (PN) remains a major limitation to treatment continuity and patient quality of life [20]. As no causal therapy for drug‐related PN currently exists, management remains limited to dose adjustments and regimen modifications aiming to mitigate severity [21]. The underlying pathomechanisms of IMiD‐induced PN and contribution of genetic susceptibility remain incompletely understood. Robust causal links have yet to be established [22]. This review cautiously evaluates current mechanistic hypotheses and genetic associations, underscoring the preliminary nature of existing evidence and methodological limitations, while proposing directions for future investigation.

## 2 Epidemiology of Peripheral Neuropathy in MM

In general, PN describes inherited or acquired damage to peripheral nerves, manifesting in sensory, motor or autonomic dysfunction, often accompanied by neuropathic pain [23, 24, 25]. MM patients carry an elevated PN risk even prior to treatment due to disease‐related mechanisms, such as paraprotein‐mediated injury, nerve compression (e.g., from extramedullary disease), and metabolic or autoimmune triggers [26]. In addition, host factors may influence treatment‐related PN risk, including diabetes, smoking, HIV/viral infections, pre‐existing neuropathy or prior neurotoxic chemotherapy and possibly obesity. Impaired renal function and vitamin B12 or folate deficiency may also increase risk, while the role of age and frailty is controversial [27, 28, 29, 30].

At first MM diagnosis, reported PN rates range from 13% to 54%, increasing to 25%–83% in relapsed or refractory settings, likely due to cumulative treatment exposure and ongoing disease activity [31, 32, 33, 34]. PN developing after treatment initiation is classified as chemotherapy‐induced PN (CIPN), commonly associated with anti‐myeloma agents such as IMiDs and Bortezomib, as well as platinum compounds and anti‐tubulins [27, 35].

### 2.1 PN Risk With Anti‐Myeloma Agents

Prescribing information and clinical trials report a wide range of PN incidence across anti‐MM agents and different combinatorial regimens (Table 1 and Table S1). Overall, Thal‐ and Bortezomib‐based treatments confer the highest incidences (up to 54% and 45%, respectively), while second‐generation IMiDs and CELMoDs demonstrate significantly lower PN rates based on prescribing information and early clinical trial data (7%–21%) [36, 37]. However, few real‐world data for Len maintenance treatment report higher PN rates up to 30%–50% [38, 39]. Fortunately, Grade 3–4 PN is very rare (< 1%) with newer IMiDs [40, 41, 42].

**TABLE 1:** Prevalence of peripheral neuropathy (PN) across anti‐myeloma drugs and regimens. Sensory and motor PN rates (all‐grade and Grade 3/4) are shown for key trials in newly diagnosed (NDMM) and relapsed/refractory multiple myeloma (RRMM). Dexamethasone (Dex).

		No. of patients	NDMM vs. RRMM	PN rate, sensory (motor, if separately reported)	
Agent	Regimen			All grades	Grade 3/4
**Monotherapy ± dexamethasone**					
Thalidomide	Thal/Dex	102 and 234	NDMM	10–54 (22)	4–5 (8)
Lenalidomide	Len maintenance	224 and 293	NDMM	15 and 10	4 and 1
	Len/Dex	353	NDMM/RRMM	7	0
Pomalidomide	Pom monotherapy	107	RRMM	21	0
	Pom/Dex	300	RRMM	17	2
Mezigdomide (CC‐92480)	Mezigdomide/Dex	77 and 101	RRMM	7–9	0–1
Iberdomide (CC‐220)	Iberdomide/Dex	90 and 107	RRMM	8–9	0–1
	Iberdomide maintenance	80	RRMM	13–15	0–3
Bortezomib	Borte monotherapy	331	RRMM	35	7
	Borte/Dex	239	NDMM	15	5
Carfilzomib	Carfilzomib/Dex	463	RRMM	12	2
Daratumumab	Dara monotherapy	2066	NDMM/RRMM	31–35	4
Belantamab mafodotin	Belantamab monotherapy	95	RRMM	NA	NA
Teclistamab	Tecli monotherapy	165	RRMM	16	0.6
Elranatamab	Elra monotherapy	183	RRMM	15.8	1.1
Talquetamab	Tal monotherapy	339	RRMM	14 (10)	0 (0.6)
Idecabtagene vicleucel (ABECMA)	Ide‐Cel	222	RRMM	10 (11)	0
Ciltacabtagene autoleucel (CARVYKTI)	Cilta‐Cel	396	RRMM	7 (13)	1 (2)
**Combination regimens**					
Bortezomib	Borte combinations	1008	RRMM	36	11
	Borte/Thal	130	NDMM	45	5
Carfilzomib	KRd	392	RRMM	11	2
Ixazomib	Ixa‐Rd	418	RRMM	28 (< 1)	2 (<1)
Isatuximab	Isa‐VRD	263	NDMM/RRMM	54	7
	Isa‐Pd/Kd	NA	NDMM/RRMM	NA	NA
Selinexor	Selinexor‐Vd	195	RRMM	32	3.6

Recent clinical trials in newly diagnosed MM using quadruplet regimens combining IMiDs and Bortezomib report consistent PN rates of 50%–60% (all grades) across risk groups (e.g., CASSIOPEIA, OPTIMUM, PERSEUS, and IMROZ) (Table S1). Despite the high overall rates, the potential synergistic neurotoxicity of IMiDs and Bortezomib remains inconclusive, as additive effects are not uniformly observed and Grade ≥ 3 PN remains unexpectedly low [43, 44]. CD38‐targeting antibodies do not appear to significantly elevate PN risk. Emerging therapies such as bispecific T‐cell engagers (BiTEs) or CAR‐T cells show consistently 10%–15% PN rates [45, 46, 47], although data remain immature. Belantamab mafodotin currently shows no evidence of PN risk.

Beyond drug‐specific effects, variability in trial‐reported PN rates likely reflects protocol heterogeneity, insensitive PN assessment tools, as well as varying exclusion criteria and incomplete documentation of pre‐existing PN rates [48]. Currently, CTCAE grading remains the clinical trial gold standard, but its limited sensitivity is expected to increase the use of composite assessment tools in the future, as already seen with the growing use of patient‐reported outcome measures [27, 49, 50].

### 2.2 Clinical Features of IMiD‐Induced PN

IMiD‐induced PN typically presents as a predominantly sensory, length‐dependent PN. This pattern likely reflects the high permeability of the blood–nerve barrier in dorsal root ganglia (DRG) sensory neurons, allowing IMiDs and metabolites to accumulate. Such accumulation may be influenced by the distinct pharmacokinetics of IMiDs (e.g., CSF penetration and hepatic metabolism). As in other CIPN, DRG injury can initiate subsequent ‘dying‐back’ axonal degeneration, with distal fibres most vulnerable due to their high metabolic demand [51, 52].

Clinically detectable PN symptoms are typically reported 2–3 months after IMiD initiation [49, 53]. Notably, subclinical PN manifestations after the first IMiD dose have been observed in nearly 100% of cases in animal models [54, 55] and one clinical trial applying rigorous assessment [33]. This initial peak appears consistent across all IMiDs, although data remain limited. The subsequent clinical course varies: Thal‐related PN symptoms typically worsen during treatment due to cumulative dose (> 20 g) and duration (> 6 months) dependency, affecting up to 70%–80% of patients at peak incidence [53, 56, 57]. Thal cessation leads to gradual improvement in most cases [49]; however, 15%–25% of patients present with irreversible symptoms [58]. Second‐generation IMiDs appear to confer less dose‐ and duration‐dependent PN risk [59]. Limited data on Len maintenance treatment suggest a stabilization of PN incidence at 10%–30% rather than a cumulative neurotoxic effect [60, 61, 62]. Such a steady state is also observed in bortezomib‐induced PN (BiPN), where symptoms typically plateau after five cycles [63]. Nonetheless, evidence remain inconclusive, ranging from reports of even PN improvement during second‐generation IMiD treatment to long‐term data in 19 MM patients showing 50% PN rates at 5 years [38].

### 2.3 Mechanistic Basis of IMiD‐Induced PN

CRBN, the primary molecular target of IMiDs, is highly expressed in the nervous system and known to be involved in neuronal signalling. However, its role in IMiD‐induced PN remains unclear, particularly since neurons do not depend on IKZF1/3 in the same way as myeloma cells.

Mechanistic insight into IMiD‐induced PN is derived from a small number of animal models in TrPN, including a rabbit model (1984, single‐agent Thal) and two rat models (2007, single‐agent Thal; 2016, VTD combination) [55, 64, 65]. Species‐specific differences between human and murine CRBN, although 94% homology, historically hampered accurate modelling in mice. In 2020, the first robust mouse model replicating IMiD‐induced neuropathic pain was established for Thal and second‐generation IMiDs as well [54].

**FIGURE 1:**
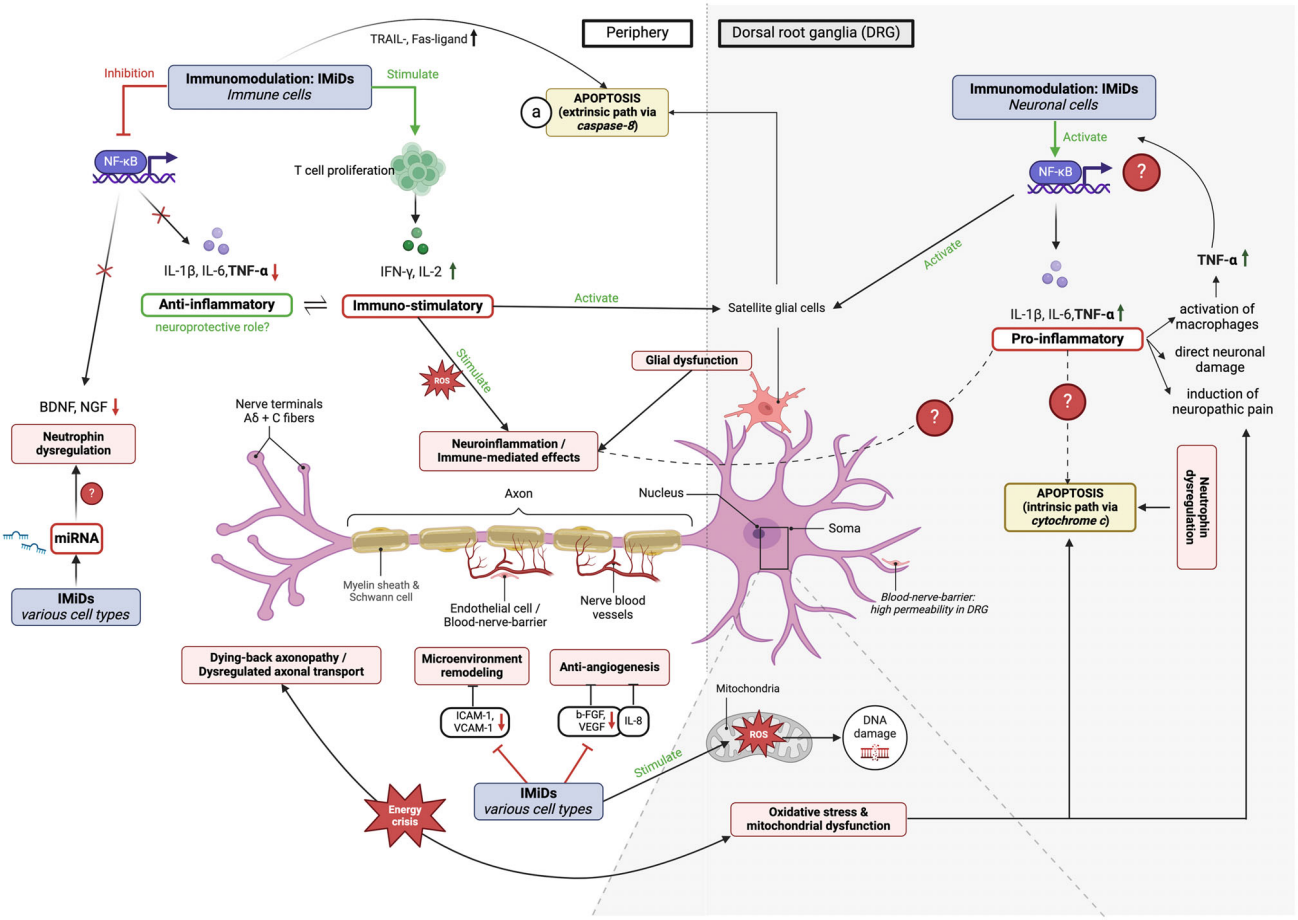
Possible mechanisms underlying IMiD‐associated peripheral neuropathy (PN). The illustration contrasts the presumed neuronal and immune‐mediated processes thought to contribute to IMiD‐associated neurotoxicity. Brain‐derived neurotrophic factor (BDNF), microRNAs (miRNA), nerve growth factor (NGF), reactive oxygen species (ROS) and tumour necrosis factor‐alpha (TNF‐α).

Beyond animal models, current pathomechanistic understanding is largely extrapolated from Thal's teratogenic mechanisms, its anti‐inflammatory properties in leprosy and known effects in immune effector cells, as well as analogies to other CIPN subtypes [9, 22]. Particularly, BiPN is of interest, since both drugs modulate NFκB‐signalling [66]. Unlike hereditary or autoimmune PN, which are commonly driven by a single dominant mechanism, IMiD‐induced PN is presumably caused by multiple overlapping mechanisms, yet poorly understood. Mainly proposed are oxidative stress, microvascular injury, neuroinflammation and neurotrophin dysregulation (Table 2, Figure 1).

**TABLE 2:** Summary of localization of neural damage and underlying pathomechanisms described in peripheral neuropathy (PN). The upper section outlines the anatomical sites at which neural injury may occur. The bottom section summarizes pathomechanisms contributing to PN, including downstream functional consequences, supporting data in the context of IMiD exposure and mechanistic examples.

Localization of damage	Consequence	Evidence IMiD‐induced PN	Common examples
Axonal damage	Distal axonal degeneration (incl. Wallerian degeneration)	No evidence	Physical nerve damage (e.g., focal compression in MM). It can be mimicked by toxins, ischaemia or inflammation
Myelin sheath/Schwann cell damage	Demyelination, impaired neural conduction	Animal model (TrPN); likely after microvascular injury	Autoimmune neuropathies (e.g., CIDP)
			Hereditary neuropathies (e.g., CMT1A—gene duplication impairs Schwann cell function)
Cell body (soma) damage	Apoptosis (intrinsic, mitochondrial pathway)	Animal model (TrPN): DRG damage	Hereditary neuropathies (e.g., spinal muscular atrophy)
			CIPN (e.g., platinum compounds form DNA adducts)

Pathomechanism		Consequence	Evidence IMiD‐induced PN	Common examples
Genetic/epigenetic factors	Genetic mutations/variation	Defective neuronal development, structure or function	Preliminary; some genetic links (TrPN)	Hereditary neuropathies (e.g., CMT subtypes, dHMN)
	microRNA dysregulation	Altered gene expression and signalling pathways	Speculative; Lenalidomide in MDS altered miRNA, no direct PN link	Diabetic PN, hereditary neuropathies
Potentially CIPN			
Functional neuronal disruptions	Altered pharmacokinetics	Drug/toxin accumulation, prolonged exposure, impaired clearance	Genetic links (TrPN), for example, ABC transporters	Polymorphisms in ADME genes
	Mitochondrial dysfunction and oxidative stress (ROS)	Energy failure and secondary neuronal damage (e.g., cell membranes, mitochondria and DNA)	Animal model (TrPN); indirect/inconclusive evidence (second‐generation IMiDs)	Most CIPN (e.g. platinum compounds, taxanes, bortezomib)
				Diabetic PN/hereditary neuropathies
	Impaired axonal transport	Disrupted delivery of organelles and proteins	Speculative; secondary to ROS	CIPN (e.g., anti‐tubulins, bortezomib)
				Hereditary PN (e.g., CMT2A—distal type)
	Neurotrophin dysregulation	Impaired neuronal survival and repair signalling	NFκB‐related; genetic link (TrPN), not directly proven	CIPN (e.g., IMiDs, bortezomib)
				Neurodegenerative diseases (e.g., Alzheimer's, ALS). They are dependent on intact retrograde axonal transport
	Ion channel dysfunction (e.g., sodium, calcium or potassium)	Altered excitability, increased neuropathic pain signalling	Neuropathic pain signalling: upregulation TRPVs (animal model, all IMiDs)	CIPN (e.g., platinum compounds, bortezomib)
				Idiopathic small fibre neuropathy, diabetic PN
	Nutritional deficiency	Impaired energy metabolism, neuronal degeneration, demyelination	Possible additive effect; unproven	Vitamin B1 (Wernicke's encephalopathy), Vitamin B12 (subacute degeneration)
Neuroimmune and microenvironmental mechanisms	Chronic inflammation	Ongoing immune activation and demyelination	No evidence	Chronic inflammatory demyelinating polyneuropathy
	Anti‐neural antibodies	Antibody‐mediated nerve injury and demyelination	No evidence	Guillain–Barré syndrome, anti‐MAG neuropathy
	Neuroinflammation	Activation of glial cells and neuropathic pain signalling	Preliminary evidence; genetic links	Neurodegenerative diseases, CIPN
	Reduced blood supply	Secondary ischaemia and hypoxia	Animal model (TrPN); indirect/inconclusive evidence (second‐ generation IMiDs)	Anti‐angiogenic agents
	Disruption of glial support	Loss of trophic and metabolic support, indirect neuronal injury	Speculative	CIPN (e.g., checkpoint inhibitors), aberrant immune responses

### 2.4 Oxidative Stress Injury

Thal's metabolites are proven to induce reactive oxygen species (ROS) [67, 68, 69], causing direct damage to DRG neurons, mitochondria and proteins, while also capable to amplify neuroinflammation (Figure 1) [70]. Ultrastructural DRG damage has been confirmed in Thal‐treated animal models [71]. Oxidative stress and associated mitochondrial dysfunction activate intrinsic apoptosis pathways, to which neurons are particularly vulnerable. Unlike immune and supporting cells, neurons express few death receptors and therefore are largely resistant to extrinsic apoptosis. Therapeutic antioxidants such as superoxide dismutase (SOD), *α*‐lipoic acid and dietary supplements (e.g., vitamins C and E, Quercetin) have been widely investigated but have shown limited efficacy in CIPN [72] (Tables S2 and S3).

Conversely, for second‐generation IMiDs, no direct neurotoxicity is proven so far, with recent data implying Pom has even antioxidative effects [7]. Finally, NFκB is a redox‐sensitive transcription factor sensible to oxidative stress [73], potentially altering tissue‐specific signalling under IMiD treatment, a concept discussed further below.

### 2.5 Microvascular Injury

It is well established that IMiDs reduce nerve blood supply via anti‐angiogenic effects (b‐FGF and VEGF inhibition) [74, 75, 76] (Figure 1). This is supported by TrPN animal models showing demyelination secondary to hypoxic injury and by the fact that therapeutic angiogenesis via intramuscular VEGF‐1 gene transfer restored vascularity and improved nerve function in established TrPN [65, 77] (Table S3). Importantly, endogenous neovascularization following microvascular damage depends on IL‐8 synthesis, which is likely suppressed by IMiD‐mediated NFκB inhibition, thus impairing vascular recovery. In addition, Thal may disrupt nitric oxide‐driven angiogenesis by inhibiting endothelial cell migration, as shown in an egg yolk model [78]. Both aspects may help explain why TrPN often improves only after Thal discontinuation.

However, the lower PN risk associated with Len and Pom, despite comparable or even greater anti‐angiogenic potency, challenges the notion that microvascular damage is the primary driver of IMiD‐induced PN. This discrepancy may result from their reduced neural accumulation due to their distinct pharmacokinetics, but interestingly, Len treatment paradoxically upregulated IL‐8 expression in MM cell cultures [79]. This would potentially support neovascularization during treatment and, although speculative, may explain the clinical improvements sometimes observed during treatment with second‐generation IMiDs.

### 2.6 Immune‐Mediated Injury and Neuroinflammation

Immune‐mediated neuronal damage and neuroinflammation are increasingly recognized as key pathomechanism in PN [80], supported by IL‐6 upregulation and TNF‐α‐mediated neurotoxicity in both clinical and preclinical CIPN models [81, 82]. The role of IMiDs in this context is controversial. While they are thought to suppress proinflammatory cytokines, suggesting rather neuroprotection, additional stress conditions could lead to the opposite, namely, NFκB‐activation and subsequent neuronal damage [79, 83]. While IMiD‐specific effects are speculative, sustained NFκB activation would promote neuroinflammation via DRG satellite glial cells (SGCs) [84]. SGCs, functionally analogous to CNS astrocytes, support DRG neurons structurally and metabolically [85] and their involvement in PN pathogenesis is suggested by observed SGC damage in bortezomib‐ and platinum‐induced PN models [86] (Figure 1). The potential context dependency of IMiD‐induced immune modulation across compartments (i.e., immune cells vs. nervous system) must be investigated to better understand their downstream effects. The contribution of other IMiD‐induced immune modulation, such as effects on macrophages, chemokine expression, T‐cell activity and IFN‐α levels, to PN pathogenesis remains unexplored to date.

### 2.7 Neurotrophin Regulation and miRNAs

**FIGURE 2:**
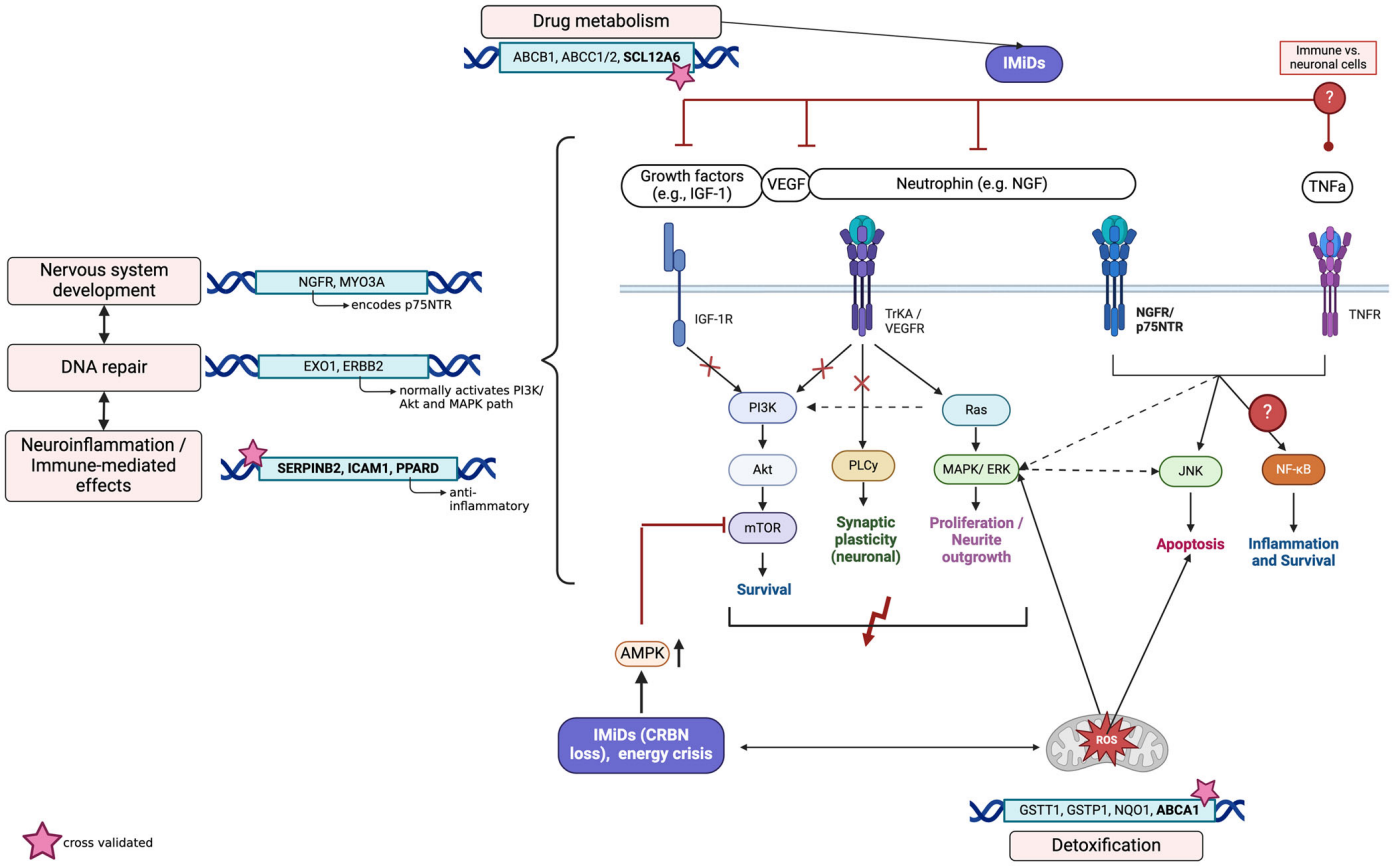
Key genes associated with thalidomide‐related peripheral neuropathy (TrPN) cluster in pathways involved in pharmacokinetics, detoxification, neurodevelopment, DNA repair and immune‐mediated/neuroinflammation. The figure highlights the principal biological pathways that are presumed to be altered in IMiD‐induced neuropathy.

Neurotrophins such as nerve growth factor (NGF) and brain‐derived neurotrophic factor (BDNF) are key regulators of neuronal survival and axonal maintenance. Impaired axonal transport and NFκB inhibition during IMiD treatment may reduce neurotrophin levels, thereby compromising neuronal survival and repair signalling [22, 87]. In addition, neurotrophin expression is regulated by microRNAs (miRNAs), which IMiDs are speculated to influence, though supporting studies remain limited. In Len‐treated del(5q) myelodysplastic syndrome (MDS), miR‐34 family downregulation was observed [8]. The miR‐34 family is involved in neuronal apoptosis and targets *EPHA5* and *SIRT1*, both implicated in PN pathogenesis [88], but a direct link to IMiD‐induced PN remains speculative. Interestingly, neurotrophin signalling converges with growth factor (e.g., VEGF and IGF‐1) and TNF‐α pathways on key intracellular mediators including PIK/Akt, MAPK, ERK1/2 and phospholipase C (Figure 2) [87]. These cascades promote NFκB‐mediated survival or apoptosis in a context‐dependent manner, making them critical for peripheral nerve repair following toxic injury [89]. Targeting these pathways has shown therapeutic promise in preclinical PN models [90] (Table S3), though no studies to date have specifically addressed IMiD‐induced PN.

### 2.8 Neuronal Hyperexcitability and Nociceptive Signalling

Neuronal hyperexcitability arises through dysregulation of voltage‐gated ion channels and transient receptor potential (TRP) channels. In peripheral nerves, sodium channel dysregulation is the major driver [91], leading to the FDA approval of the first‐in‐class NaV1.8 inhibitor (suzetrigine) for neuropathic pain in 2024 [92]. Both oxidative stress and proinflammatory cytokines further amplify nociceptive signalling (Figure 1) [81, 93], in part via ROS‐induced upregulation of TRPA1 and TRPV4, as shown in animal models treated with Thal, Len or Pom [54, 94].

While IMiDs may sensitize nociceptive pathways under certain conditions, contributing to neuropathic pain, their anti‐inflammatory properties have conversely demonstrated analgesic effects in CIPN models, for example, when administering Thal intrathecally in BiPN. Consistent with this, TNF‐α blockade has shown benefit in pain alleviation [95, 96, 97]. This duality may help explain the inconsistent evidence for additive neurotoxicity when IMiDs are combined with bortezomib [44].

### 2.9 Role of Genetics in IMiD‐Induced PN

Mechanistic uncertainties and substantial interpatient variability in the incidence and severity of IMiD‐induced PN remain major challenges. Emerging data now suggest that genetic factors may contribute to individual PN risk [98, 99]. Interestingly, the CASSIOPEIA trial reported divergent PN rates between countries (33% in France vs. 49% in the Netherlands) despite similar protocols [100], and patients with hereditary neuropathies such as Charcot–Marie–Tooth (CMT) disease show in general increased CIPN susceptibility [101, 102]. Although mechanistic evidence remains sparse, several pharmacogenomic studies over the past decade have identified 72 genes potentially linked to TrPN risk (Table S4). No genetic association study is currently available for PN related to second‐generation IMiDs.

In the following section, 15 genes with the strongest biological plausibility and/or consistent replication in independent cohorts are discussed (Table 3). A polymorphism in the CRBN promoter was linked to increased TrPN risk [103], possibly through altered AMPK/mTOR signalling, which is observed in a hippocampal *CRBN* knockout mice model [104]. Other genes associated to TrPN risk cluster in pathways related to pharmacokinetics, detoxification, neurodevelopment, DNA repair and inflammation (Figure 2) [105, 106, 107, 108]. Genetic variation in pharmacokinetics, particularly in genes involved in absorption, distribution, metabolism and excretion (ADME), has been repeatedly observed. Single nucleotide polymorphisms (SNPs) in efflux transporters (e.g., *ABCB1*, *ABCC1*, and *ABCC2*) and axonal cotransporters (e.g., *SLC12A6*) have been associated with increased TrPN risk (Table 3). Of note, the pathomechanistic role of *SLC12A6* has been validated in two MM trials (HOVON‐50, Myeloma IX) and is also implicated in hereditary neuropathies (i.e., CMT‐4) [109] (Figure 2).

**TABLE 3:** Summary of genes implicated in thalidomide‐related peripheral neuropathy (TrPN), including gene function, associated regimens, supporting evidence and PubMed references.

Gene	Function	Treatment regimen	Validation in TrPN/association to other PN	PMID
*ABCA1*	ADME—lipid homeostasis	CTD/TAD	Validated in independent MM cohort	21245421
*ABCB1* (*MDR‐1*)	ADME—efflux transporter	CTD	Paclitaxel‐induced PN	21245421, 16950614
*ABCC1*		CTD	Vincristine‐induced PN, late BiPN	21245421, 20864405, 21791469
*ABCC2*		CTD	Taxane‐induced PN	21245421, 24599932
*ERBB2 (HER2/neu)*	Cell growth, differentiation, neuronal survival	CTD		21245421
*EXO1*	DNA repair	CTD	Early BiPN	21245421, 20864405
*GSTP1*	Detoxification, oxidative stress defence	CTD	Platin‐induced PN	21245421, 16707601
*GSTT1*		Thal mono		21435719
*ICAM1*	Immune system regulation (cell adhesion, inflammation)	CTD/TAD	Validated in independent MM cohort	21245421, 28817461
*IGF1R*	Cell growth and survival, nervous system development (neurotrophic signalling)	CTD	Early BiPN	21245421, 20864405
*NGFR*	Neuronal survival and regeneration	CTD	BiPN, hereditary PN	21245421, 21791469
*NQO1*	Detoxification, oxidative stress defence	CTD		21245421
*PPARD*	Energy metabolism, inflammation regulation, transcription factor	CTD / TAD	Validated in independent MM cohort, vincristine‐induced PN, late BiPN	21245421, 20864405
*SERPINB2 (PAI‐2)*	Inflammation regulation (plasminogen activator inhibitor)	CTD/TAD, Thal mono	Validated in independent MM cohort, late BiPN	21245421, 28817461, 20864405
*SLC12A6*	ADME—potassium‐chloride cotransporter (KCC3)	CTD/TAD	Validated in independent MM cohort, early vincristine‐induced, hereditary PN	21245421, 20864405

Abbreviation: BiPN; Bortezomib‐induced PN.

Polymorphisms in detoxification genes such as *GSTT1*, *GSTP1* and *NQO1* as well as in DNA repair genes (e.g., *EXO1* and *ERBB2*) were also linked to TrPN susceptibility, though mechanistic validation remains lacking. Genes involved in neuroinflammation (e.g., *SERPINB2*, *ICAM1*, and *PPARD*) accounted for around 35% of TrPN‐associated SNPs in the landmark study conducted by Johnson et al. [106]. *SERPINB2* and *ICAM1* could be validated across MM cohorts (HOVON‐50 trial) and other disease settings (inflammatory bowel disease) [108] (Table 3), making them strong candidates for true biological contributors.

Furthermore, SNPs in *NGFR*, encoding the neurotrophin receptor p75NTR, could point to the pathomechanistic role of neurotrophin signalling in TrPN. *NGFR* polymorphism is also proven in hereditary PN (i.e., HSAN‐5) [110] (Table 3). Finally, gene expression data from MM plasma cells are scarce in the context of IMiD‐induced PN, suggesting possible involvement of cytoskeletal and neurodevelopmental pathways in affected patients [44]. However, this is tempered by the lack of corresponding data from neural tissue.

Overall, while genetic susceptibility appears plausible, current evidence is preliminary, limited by small cohorts, inconsistent replication and lack of functional validation, particularly with respect to second‐generation IMiDs, where genetic data are absent.

### 2.10 Clinical Management of IMiD‐Induced PN

In the absence of a causal mechanism to date, there is no specific therapy for IMiD‐induced PN. Current clinical management typically involves dose adjustments or regimen changes [21], with supportive measures like vitamin supplementation, and lifestyle modifications offering uncertain benefits [72] (Tables S2 and S3). The 2024 approval of first‐in‐class NaV1.8 inhibitor (suzetrigine) may aid neuropathic pain management, but its efficacy in IMiD‐induced PN is presently untested and might be influenced by IMiD‐specific upregulation of TRPV. Interventions like cryotherapy, showing promise in other CIPN [111], have not yet been evaluated, and clinical trials focusing IMiD‐specific PN are notably absent.

## 3 Discussion

### 3.1 Neurotoxicity: Thalidomide Versus Second‐Generation IMiDs

Thal undoubtedly confers a higher neurotoxic risk than second‐generation IMiDs. Robust preclinical and clinical data in TrPN implicate direct neuronal injury, driven by oxidative stress and microvascular damage. These conditions possibly promote subsequent neuroinflammatory cascades (i.e., NFκB activation) and contribute to sustained damage. Since Thal likely inhibits IL‐8, thereby impairing neovascularization, regenerative capacity appears to resume post‐discontinuation in most patients, although some show irreversible nerve damage [58].

Interestingly, second‐generation IMiDs, despite stronger CRBN binding, NFκB/TNF‐α inhibition and enhanced anti‐angiogenic potency, appear to be associated with significantly lower PN incidence. Several mechanisms may contribute (1) enhanced immunomodulatory selectivity and promotion of IL‐10, a cytokine essential for neuronal repair [112]; (2) preserved IL‐8‐mediated neovascularization during treatment, which may protect against hypoxia‐related injury observed with Thal; (3) reduced expression of adhesion molecules, potentially limiting immune cell infiltration and stabilising the blood–nerve barrier; and (4) favourable pharmacokinetics, such as lower tissue accumulation and reduced CSF penetration [19].

The hypothesis would be that under conditions of minimal additional cellular stress and preserved clearance capacity, second‐generation IMiDs, particularly Pom, may even confer neuroprotection [113]. This is supported by early studies in neurodegeneration models [7, 114, 115]. However, clinical and mechanistic data remain inconsistent, and long‐term outcomes are poorly studied. For CELMoDs, early‐phase clinical trials suggest similarly low, if not lower, rates of PN [36, 37, 116, 117]. However, mechanistic data are lacking and clinical follow‐up remains immature. Thus, any neuroprotective potential of newer IMiDs remains hypothetical and must be evaluated cautiously.

### 3.2 Hypothesized Temporal Dynamics of IMiD‐Induced PN

**FIGURE 3:**
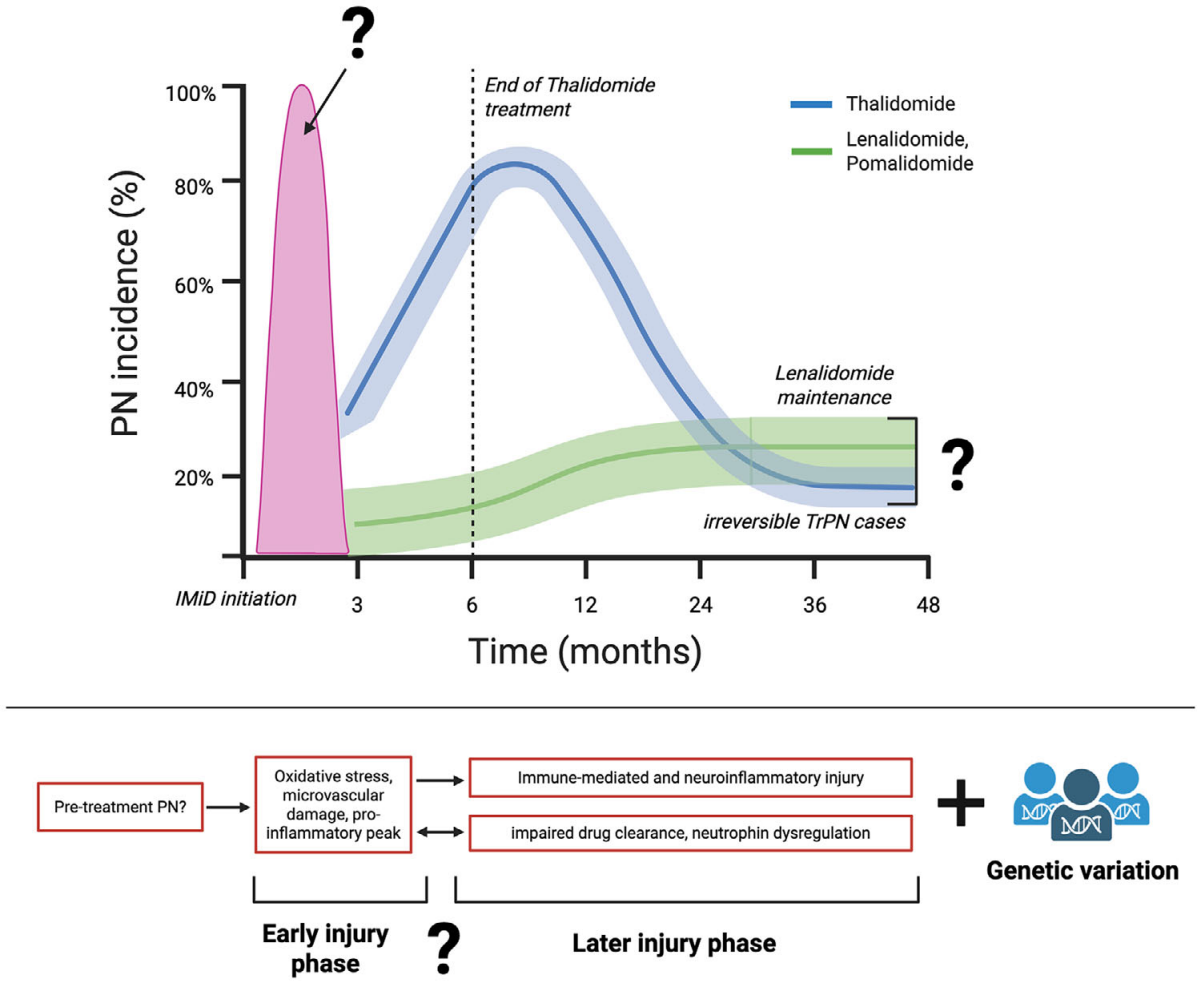
Suggested temporal dynamics of IMiD‐induced peripheral neuropathy (PN) with thalidomide and second‐generation IMiDs. Based on preclinical and limited clinical data 33, 54, 55, we propose that IMiD‐related PN may follow a biphasic course, comprising early and late phases of neuronal injury driven by distinct dominant biological processes. The early phase may primarily reflect acute neuronal injury mediated by oxidative stress and microvascular dysfunction. With ongoing therapy, PN may evolve into a more complex immune‐mediated and neuroinflammatory injury state. This later phase may be more strongly influenced by additional co‐stressors and could determine whether patients recover or develop progressive PN symptoms. The severity and trajectory of both phases might be further modulated by individual genetic variation.

IMiD‐induced PN may follow a biphasic course (Figure 3). As observed in BiPN, distinct molecular profiles between early‐ and late‐onset PN suggest temporally dynamic pathomechanisms [89]. A concept that may extend to IMiD‐induced PN, although current evidence remains speculative. The early phase probably reflects acute neuronal injury driven by oxidative stress and microvascular dysfunction. Preclinical and limited clinical trial evidence suggest such subclinical injury may occur in up to 100% of patients [33, 54, 55]. While recovery is typical after single‐dose IMiD exposure in animal models, ongoing therapy may lead to a more complex, immune‐mediated and neuroinflammatory injury, as suggested above for TrPN. Probably, this second phase is more strongly modulated by additional co‐stressors and determines whether patients recover or show progressive PN symptoms.

Given the variability in PN incidence, and the fact that some patients experience substantial neurotoxicity even with second‐generation IMiDs, it would be plausible that temporal PN dynamics are modulated by genetic susceptibility, individually affecting early or late phases. Variants affecting drug clearance (e.g., *ABCB1*), detoxification (e.g., *GSTT1*), inflammation control (e.g., *SERPINB2, ICAM1*) or DNA repair (e.g., *EXO1, ERBB2*) may shift NFκB signalling in neuronal tissue toward activation, thereby sustaining neuroinflammation and exacerbating neuronal injury. Early injury prevention and modulation of the co‐stressors (e.g., local antioxidative or anti‐inflammatory effects) could thus be critical for long‐term outcomes.

### 3.3 Challenges and Future Directions

Research faces significant hurdles in establishing causality for IMiD‐induced PN [118]. Preclinical models are limited, with only one recent murine model providing partial insights, and key neuronal tissue‐specific mechanisms remain uncharacterized. Clinical and genetic association studies are hampered by small cohorts, phenotypic heterogeneity and insensitive diagnostic tools [49, 119]. Frequent exclusion of patients with baseline PN ≥ Grade 2 and underreporting of pre‐treatment PN rates further compromise data interpretation. In a meta‐analysis of nine CIPN‐related GWAS, Mahmoudpour et al. found replication success only in studies with > 300 participants [120]. In line, García‐Sanz et al. identified no TrPN‐associated variant unless analysing 300,000 exome SNPs in 172 MM patients [121]. These limitations highlight the need for standardized phenotype definitions and adequately powered cohorts (> 300 patients). Moreover, second‐generation IMiD‐induced PN remains poorly understood, both in terms of their clinical course and the absence of genetic association studies to date. Ongoing studies such as CIPN‐REBECCA (NCT06052345), which employ wearable sensors for early detection of paclitaxel‐induced PN, may offer transferable insights and should be considered for adaptation in MM cohorts receiving IMiD‐based therapies.

These limitations highlight key considerations for future trials of second‐ and next‐generation IMiDs. These should include standardized phenotyping, systematic assessment of pre‐treatment PN and host risk factors, serial patient‐reported and CTCAE‐based assessments, predefined sensory and motor PN endpoints, prospective documentation of PN‐related treatment modifications and reversibility, as well as collection of germline and pharmacokinetic data.

Preclinical research must address IMiD‐specific effects on neural tissue, potentially leveraging models of hereditary neuropathies (e.g., CMT‐4 and HSAN‐5). The context‐specific activity of IMiDs, such as NFκB signalling and CRBN substrate modulation, remains an unmet research need. Emerging data on alternative CRBN substrates (e.g., CSNK1A1 in del(5q) MDS treated with Len [122]) suggest potential neural‐specific substrates that could influence neurotoxicity, though direct evidence is lacking.

Finally, therapeutic exploration is equally warranted. Regenerative approaches, such as mesenchymal stem cell‐derived neural precursors (under evaluation in CMT‐1), may hold promise. Adjunctive agents like statins, which have been shown to reduce PN in diabetic models via antioxidative and anti‐inflammatory effects [123], may complement IMiD therapy in reducing co‐stressors. Harnessing IMiD‐induced immunomodulation to counteract neurotoxicity in T‐cell‐directed therapies is an interesting concept, considering that full PN resolution was observed in only 52% of cases in CAR‐T trials [45]. While promising, these directions remain hypothesis‐generating and require robust mechanistic data to enable therapeutic translation.

## 4 Conclusion

IMiD‐induced PN poses a complex challenge in MM therapy, with suggested mechanisms including oxidative stress, microvascular changes, neuroinflammation and genetic factors, potentially modulated by IMiD pharmacokinetics. Thal's neurotoxicity contrasts with the possibly lower risk associated with Len and Pom, but evidence is limited, variable and insufficient to confirm causality. Methodological constraints, such as small studies and inconsistent phenotyping, highlight the need for standardized approaches, larger genetic cohorts and improved preclinical models to cautiously advance understanding and management of this toxicity, ultimately supporting patient quality of life.

## Author Contributions

**Sina A. Beer**: writing – original draft. **Richard Houlston**: writing – review and editing. **Martin F. Kaiser**: writing – review and editing. **Sina A. Beer**: visualization.

## Funding

The authors have nothing to report.

## Ethics Statement

The authors have nothing to report.

## Consent

The authors have nothing to report.

## Conflicts of Interest

Martin F. Kaiser received consultancy and honoraria from AbbVie; research funding from BMS; consultancy from GSK; research funding, consultancy and honoraria from Janssen; and consultancy from Karyopharm, Pfizer, Regeneron and Takeda. The other authors declare no conflicts of interest.

## Supporting information




**Supporting Information**: jha270323‐sup‐0001‐tableS1‐S4.xlsx" alt

## Data Availability

The authors have nothing to report.
